# Multimorbidity patterns in South Africa: A latent class analysis

**DOI:** 10.3389/fpubh.2022.1082587

**Published:** 2023-01-11

**Authors:** Rifqah Abeeda Roomaney, Brian van Wyk, Annibale Cois, Victoria Pillay van-Wyk

**Affiliations:** ^1^Burden of Disease Research Unit, South African Medical Research Council, Cape Town, South Africa; ^2^School of Public Health, University of the Western Cape, Cape Town, South Africa; ^3^Division of Health Systems and Public Health, Department of Global Health, University of Stellenbosch, Stellenbosch, South Africa

**Keywords:** multimorbidity, disease patterns, disease clusters, latent class analysis, prevalence, South Africa

## Abstract

**Introduction:**

South Africa has the largest burden of HIV worldwide and has a growing burden of non-communicable diseases; the combination of which may lead to diseases clustering in ways that are not seen in other regions. This study sought to identify common disease classes and sociodemographic and lifestyle factors associated with each disease class.

**Methods:**

Data were analyzed from the South African Demographic and Health Survey 2016. A latent class analysis (LCA) was conducted using nine disease conditions. Sociodemographic and behavioral factors associated with each disease cluster were explored. All analysis was conducted in Stata 15 and the LCA Stata plugin was used to conduct the latent class and regression analysis.

**Results:**

Multimorbid participants were included (*n* = 2 368). Four disease classes were identified: (1) HIV, Hypertension and Anemia (comprising 39.4% of the multimorbid population), (2) Anemia and Hypertension (23.7%), (3) Cardiovascular-related (19.9%) and (4) Diabetes and Hypertension (17.0%). Age, sex, and lifestyle risk factors were associated with class membership. In terms of age, with older adults were less likely to belong to the first class (HIV, Hypertension and Anemia). Males were more likely to belong to Class 2 (Anemia and Hypertension) and Class 4 (Diabetes and Hypertension). In terms of alcohol consumption, those that consumed alcohol were less likely to belong to Class 4 (Diabetes and Hypertension). Current smokers were more likely to belong to Class 3 (Cardiovascular-related). People with a higher body mass index tended to belong to Class 3 (Cardiovascular-related) or the Class 4 (Diabetes and Hypertension).

**Conclusion:**

This study affirmed that integrated care is urgently needed, evidenced by the largest disease class being an overlap of chronic infectious diseases and non-communicable diseases. This study also highlighted the need for hypertension to be addressed. Tackling the risk factors associated with hypertension could avert an epidemic of multimorbidity.

## Introduction

Multimorbidity (living with more than one chronic disease) is associated with an increased risk of mortality (1), poorer self-rated health (2), reduced quality of life and increased healthcare utilization and associated costs (3). The prevalence of multimorbidity is likely to increase as populations' age, the burden of non-communicable diseases (NCDs) grows (4), and chronic complications arise due to infections from COVID-19. Approximately, 30% of people in low and middle income countries (LMICs) are living with multimorbidity (5). Still, compared to high income countries, much less is known about multimorbidity in LMICs; with LMICs accounting for only 5% of the scientific literature on multimorbidity globally (6). An increase in the prevalence of multimorbidity could prove dire to many countries already struggling to cope with the ill health of their populations.

Multimorbidity is thought to start at younger ages in LMICs (4), likely due to disease burdens affecting younger people such as HIV. With the increased availability of antiretroviral therapy (ART), life expectancies for people living with HIV have increased (7). HIV is now considered a chronic disease and is commonly co-morbid with chronic diseases such as hypertension, dyslipidaemia, diabetes, and cardiovascular disease (8).

South Africa has one of the highest HIV prevalence in the world, with 7.9 million people living with HIV in 2017 (9). The HIV prevalence reaches 33.3% in females and 19.4% in males between the ages of 25–49 years (9). South Africa boasts the largest ART programme in the world, with 5,599,664 adults and children on ART (10). Furthermore, South Africa reports high burdens of disease due to tuberculosis (TB), NCDs, injuries, and maternal and child health (11). A systematic review of multimorbidity in the country (12) found that multimorbidity is prevalent, especially among women and older adults.

While knowing the prevalence of multimorbidity is important, it is also vital to understand how diseases cluster together and what factors are associated with the clustering. This can better enable researchers and clinicians to develop appropriate guidelines for the management of multimorbidity, generate new hypotheses on etiology underlying associations, facilitate studies to identify risk factors (7) and identify groups of people to target for screening interventions. Latent class analysis (LCA) is a popular method used to identify subgroups or classes (13). LCA is a cross-sectional latent variable mixture modeling technique which aims to find heterogeneity within the population and probabilistically assigns each individual to a class (13). LCA is considered advantageous over other clustering techniques as it provides fit statistics and covariates can be included in models (13).

LCA has been used to identify multimorbidity disease patterns or classes in several other studies. A recent study analyzed NCD data to determine latent classes in older South Africans and identified three groups, namely: minimal multimorbidity risk (83%), concordant multimorbidity (11%) and discordant multimorbidity (6%) (14). Similarly, our study aims to determine disease patterns among multimorbid people in South Africa using LCA. Where our study differs is that we limited our analysis to multimorbid people, we used a more inclusive age range (15 years and older) and we included chronic infectious diseases such as HIV, which is important to the local context. We aimed to determine sociodemographic and lifestyle factors associated with each disease class. To our knowledge, this is the first South African LCA study that includes HIV in an analysis of multimorbidity patterns.

## Materials and methods

### Data, measures of disease and variables of interest

This study used data from a nationally representative survey, the South African Demographic and Health Survey (SADHS) 2016. The survey used a stratified two-stage sample design and a total of 750 primary sampling units were selected. Twenty dwelling units were randomly selected in each primary sampling unit and these were sub-sampled such that half of the households were eligible for a South African-specific module on adult health that included the collection of biomarkers (15). Detailed methods can be found elsewhere (15). This analysis was restricted to persons 15 years and older who had more than one disease condition.

We included disease conditions which could be deemed to be “current.” Two clinicians assisted where the information was unclear. Individuals were asked whether they were diagnosed by a health worker with the following conditions: diabetes, heart disease, high blood cholesterol, stroke, TB in the last 12 months and chronic obstructive pulmonary disease (COPD) or bronchitis.

For testing HbA1c and HIV, nurses collected finger-prick blood specimens on a filter paper card. The dry blood spot for HbA1c was analyzed with a blood chemistry analyser measuring total hemoglobin levels (15). The presence of diabetes was indicated either by the presence of an HbA1c level >6.5 mmol/l (16, 17), through self-report, or if a participant was on treatment for diabetes. For HIV, dry blood spots were tested with enzyme-linked immunosorbent assay (ELISA) and confirmed with a second test (15). We included the results of the first ELISA.

Blood specimens for anemia testing were collected in a microcuvette and hemoglobin levels were tested on-site to detect the presence or absence of anemia (15). We considered anemia to be present whether mild (pregnant women: Hg levels between 10.0–10.9 g/dl, other adults: 10.0–11.9 g/dl), moderate (7.0–9.9 g/dl) or severe (<7.0 g/dl) (18). Participants had their blood pressure measured three times and we averaged the second and third measurements (19). Hypertension was defined as having blood pressure outside the health range (Stage 1 Hypertension: systolic: 140–159 mmHg or diastolic: 90–99 mmHg; Stage 2 Hypertension: Systolic ≥160 mmHg or diastolic ≥100 mmHg) or being on antihypertensive medication (20). Further details on data cleaning are provided in Supplementary Table S1.

Self-reported demographic variables included age, sex, and years of schooling (primary completed, secondary completed and tertiary education) (15). Behavioral and lifestyle variables were also included based on factors commonly associated with multimorbidity (6). We determined current alcohol use (in the past 12 months) by combining the responses to two questions (“Have you ever consumed a drink that contains alcohol such as beer, wine, ciders, spirits, or sorghum beer?” and “Was this within the last 12 months?”). For tobacco use, those that smoked either every day or some days were considered to be current smokers. Body Mass Index (BMI) was also considered. Participants had their height and weight measurements taken. BMI was categorized as underweight, normal weight, and overweight/obese.

Participants gave their consent to take part in the survey, take measurements of heights, weights and blood pressure and collect blood specimens. This study was a secondary data analysis of an anonymised dataset which was obtained from the DHS programme. Ethics clearance to conduct this study was granted by the Biomedical Research Ethics Committee of the University of the Western Cape (BM20/5/8) as part of the author's Ph.D. project.

### Statistical analysis

Data cleaning was conducted in Stata 15 (College Station, TX: StataCorp LLC). The LCA Stata Plugin (21) was used as it accommodates clustering and weighting common to surveys with complex sampling designs. Nine disease outcomes were included as indicator variables and were coded as binary variables (i.e., disease present or disease absent).

Bivariate data analysis was conducted to describe the multimorbid population by sex. Age was analyzed as a continuous variable and the median and interquartile range was calculated. The association between age and sex was calculated using a Wilcoxon rank sum test. Locality, province, educational level, employment, and wealth index were analyzed as categorical variables. The association of these variables with sex was tested using Chi-squared tests.

Initially, the model selection for the LCA was done without covariates (22). We first estimated a one-class model and then added additional classes to compare the relative fit of each model using fit statistics (22). We compared the Bayesian information criterion (BIC) (23), the Akaike information criterion (AIC) (24) and the adjusted BIC (aBIC) (25). We determined which model had the lowest AIC, BIC, and aBIC values (with lower values indicate a better relative fit). The substantive meaning of the classes were also considered i.e., whether the classes are what would be expected from theory, whether they are easy to interpret and also whether classes are large enough (13). Once the appropriate LCA model was selected, each individual was assigned to a class based on their posterior class membership probabilities. The LCA Stata Plugin contains multinomial logistic regression options for predicting latent class membership (21). Using the LCA Stata Plugin, we performed a multivariate regression with class membership as the outcome. Covariates were investigated based on factors that are commonly associated with multimorbidity (26). These included age, sex, locality (rural/urban), educational attainment, BMI, and alcohol drinking and tobacco smoking status. We also explored employment status and wealth index but these were later removed due to the sparse design matrix. A reference class was specified by the researchers and was based on which class was the largest. We chose to exclude participants that were not multimorbid as a large number of “healthy” individuals would present problems in detecting multimorbid classes.

## Results

Of the 2,368 multimorbid participants, the majority were female (71.5%) (Table 1). The median age was 50 years. Generally, males and females were similar in most aspects, but males were significantly more likely to be employed and were more likely to belong to wealthier quintiles.

**Table 1:** Description of the multimorbid population by sex (unweighted).

	**Total (*N* = 2,368)**	**Male (*N* = 674)**	**Female (*N* = 1,694)**	***p*-value^a^**
	**% (*n*)**	**% (*n*)**	**% (*n*)**	
Age^*^ (median years and IQR)	50 (37–63)	52 (39–64)	50 (36–63)	0.527
Urban location	53.7 (1,271)	55.6 (375)	52.9 (896)	0.227
**Province**				0.532
Western Cape	6.9 (163)	7.3 (49)	6.7 (114)	
Eastern Cape	15.9 (376)	15.1 (102)	16.2 (274)	
Northern Cape	7.3 (172)	8.9 (60)	6.6 (112)	
Free State	13.6 (323)	13.2 (89)	13.8 (234)	
Kwa-Zulu Natal	16.4 (389)	16.6 (112)	16.4 (277)	
North West	10.5 (249)	11.1 (75)	10.3 (174)	
Gauteng	7.1 (169)	7.7 (52)	6.9 (117)	
Mpumalanga	13.2 (313)	11.9 (80)	13.8 (233)	
Limpopo	9.0 (214)	8.2 (55)	9.4 (159)	
**Education level**				0.132
Primary or less	40.2 (953)	40.1 (270)	40.3 (683)	
Secondary complete	53.5 (1,266)	52.1 (351)	54.0 (915)	
Tertiary	6.3 (149)	7.9 (53)	5.7 (96)	
Employed	30.7 (727)	38.9 (262)	27.4 (465)	**< 0.001**
**Wealth index**				**0.002**
Quintile 1 (poorest)	20.3 (481)	20.9 (141)	20.1 (340)	
Quintile 2 (poorer)	21.2 (503)	21.5 (145)	21.1 (358)	
Quintile 3 (middle)	23.8 (563)	21.2 (143)	24.8 (420)	
Quintile 4 (richer)	20.9 (494)	18.4 (124)	21.8 (370)	
Quintile 5 (richest)	13.8 (327)	18.0 (121)	12.2 (206)	

* Age in years.

a Categorical variables tested using Chi-squared test, continuous variables tested using Wilcoxon rank sum test. Bold values are used to represent where *p* < 0.001.

### Distribution of diseases in the multimorbid population

**Figure 1:**
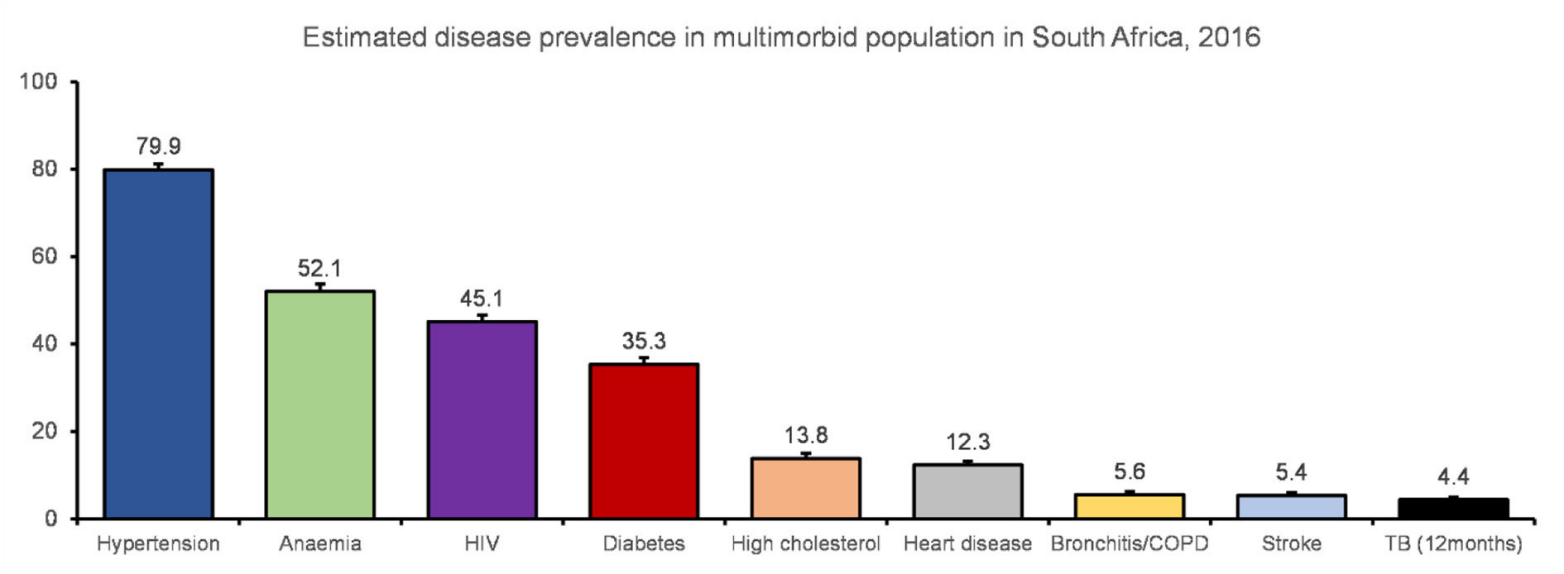
Estimated disease prevalence in the multimorbid population (weighted).

Among the population with multimorbidity, the majority were estimated to have hypertension (Figure 1). Anemia, HIV and diabetes were also prevalent. When taking age into account, hypertension, diabetes, heart disease, high cholesterol, bronchitis/COPD and stroke increased in older age groups (Supplementary Figure S1). Most of the disease conditions were more prevalent among females compared to males (Supplementary Table S2).

Table 2 shows a comparison of fit statistics for models with different numbers of classes, ranging from two to seven. The BIC and adjusted BIC were minimal for 4 and 5 classes, respectively. The AIC suggested a larger number of classes, but the AIC tends to suggest overly complex models (22). Using 5 rather than 4 classes produced only a modest decrease in the aBIC but did not improve clinical interpretability (e.g., the “Cardiovascular class” of diseases was further split into smaller groups), hence, we selected a 4-classes model as the optimal one. We adopted class names based on either the disease/s with the highest probability or categories of clinical significance.

**Table 2:** Fit statistics for LCA models with different numbers of classes.

**Fit statistics**	**Latent class analysis models**					
	**2 class model**	**3 class model**	**4 class model**	**5 class model**	**6 class model**	**7 class model**
Design effect	1.8	1.7	1.5	1.6	1.4	1.4
Degrees of freedom	492	482	472	462	452	442
Entropy R-squared	0.8	0.9	0.8	0.9	0.9	0.9
Entropy raw	337.5	370.8	566.0	565.6	553.3	489.8
Adjusted BIC	1,699.8	1,359.5	1,160.7	1,135.1	1,159.5	1,158.7
BIC	1,760.2	1,451.6	1,284.6	1,290.8	1,347.0	1,377.9
AIC	1,650.5	1,284.3	1,059.5	1,008.1	1,006.6	979.8
G-squared	1,612.5	1,226.3	981.5	910.1	888.6	841.8
Log likelihood	−8,343.2	−8,150.1	−8,027.7	−7,992.0	−7,981.2	−7,957.9

**Figure 2:**
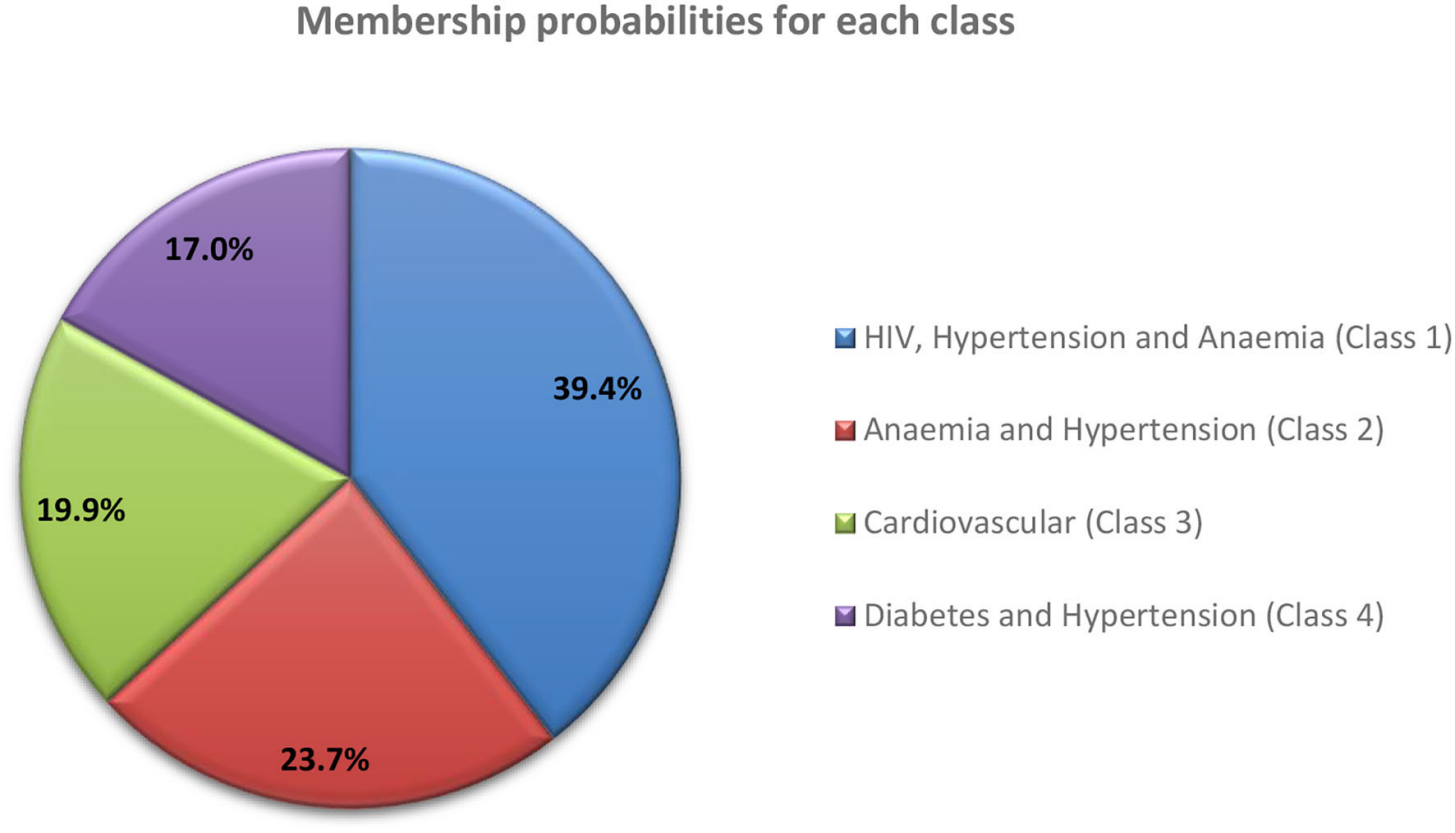
Membership probabilities for each latent class.

The reported membership probability represents the “conditional prevalence” for each retained class or the estimated distribution of the multimorbid population across the four latent classes. Four disease classes identified and membership probabilities are shown in Figure 2 (standard errors available in Supplementary Table S3). Class 1 (HIV, Hypertension and Anemia) accounted for almost 40% of the multimorbid population.

Class 1 *(HIV, Hypertension and Anemia)* was characterized by HIV (Table 3). It was predicted that all members have HIV, 61% hypertension, 59% anemia and other diseases in smaller percentages. Class 2 (*Anemia and Hypertension*) was characterized by anemia, with all members predicted to have anemia, 87% hypertension, followed by the other diseases in smaller percentages. For Class 3 (*Cardiovascular*), 93.8% of members were predicted to have hypertension, 50% high cholesterol, 38% heart disease, 35% diabetes, followed by the other diseases in smaller percentages. Class 4 (*Diabetes and Hypertension*), was characterized by all members predicted to have diabetes and hypertension, followed by the other diseases in smaller quantities. The item response probabilities with standard errors are shown in Supplementary Table S4.

**Table 3:** Item response probabilities, by disease for each latent class.

**Class name**		**Disease condition (Item response probabilities)**								
		**Anemia**	**Bronchitis/** **COPD**	**Diabetes**	**Heart disease**	**High cholesterol**	**HIV**	**Hypertension**	**Stroke**	**TB** *
1	HIV, hypertension and anemia	**0.59**	0.01	0.10	0.05	0.01	**1.00**	**0.61**	0.02	0.08
2	Anemia and hypertension	**1.00**	0.03	0.31	0.08	0.04	0.00	**0.87**	0.03	0.02
3	Cardiovascular	0.17	0.22	0.35	0.38	**0.50**	0.08	**0.94**	0.18	0.05
4	Diabetes and hypertension	0.00	0.00	**1.00**	0.06	0.14	0.09	**1.00**	0.03	0.01

* TB in the last 12 months.

Probabilities >50% are bolded.

Class names are based on either the disease/s with the highest probability or categories of clinical significance.

### Factors associated with latent class membership

The latent class model was run with covariates to identify associations with a membership of the four latent classes (Table 4, Supplementary Tables S5, S6). Class 1 (*HIV, Hypertension and Anemia*) was selected as the reference class because it was the largest disease class identified in our study. Age was significantly associated with class membership. Older adults (people aged 55+ years) were more likely to belong to the three classes compared to Class 1. Sex was associated with two of the classes i.e., males were more likely to belong to the *Anemia and Hypertension* class or the *Diabetes and Hypertension* class, compared to Class 1.

**Table 4:** Factors associated with class membership.

**Covariates**	**OR (95% CI) by class membership**			
	**Class 1** **(Reference class)**	**Class 2**	**Class 3**	**Class 4**
	**HIV, hypertension and anemia**	**Anemia and hypertension**	**Cardiovascular**	**Diabetes and hypertension**
**Age category (reference: 15–34 years)**				
35–54 years	1.00	1.189	**4.533**	**4.12**
		(0.842–1.679)	**(2.398–8.566)**	**(2.582–6.572)**
55+ years	1.00	**4.607**	**61.744**	**25.352**
		**(2.987–7.107)**	**(30.169–126.364)**	**(14.09–45.614)**
Female (reference: male)	1.00	**0.613**	1.006	**0.593**
		**(0.429–0.876)**	(0.652–1.553)	**(0.417–0.842)**
Urban location (reference: rural)	1.00	1.048	**1.91**	0.755
		(0.763–1.439)	**(1.243–2.937)**	(0.53–1.076)
**Education (reference: none/primary)**				
Secondary education	1.00	0.819	1.393	0.921
		(0.609–1.101)	(0.922–2.105)	(0.675–1.255)
Tertiary education	1.00	**2.448**	**5.588**	**2.517**
		**(1.367–4.384)**	**(2.909–10.734)**	**(1.481–4.275)**
Currently drinks alcohol (reference: not current drinker)	1.00	0.826	1.106	**0.58**
		(0.581–1.174)	(0.719–1.702)	**(0.41–0.822)**
Currently smokes (reference: not current smoker)	1.00	0.984	**1.681**	1.388
		(0.623–1.554)	**(1.038–2.721)**	(0.937–2.055)
**BMI (reference: normal weight)**				
Underweight	1.00	0.891	1.96	**0.492**
		(0.428–1.857)	(0.868–4.422)	**(0.28–0.865)**
Overweight/obese	1.00	1.184	**2.902**	**3.934**
		(0.834–1.682)	**(1.757–4.791)**	**(2.852–5.427)**

Class 1 (HIV, hypertension and anemia) is the reference category. Bold values are used to represent where *p* < 0.001.

Those with tertiary education were more likely to belong to the *Anemia and Hypertension* class, the *Cardiovascular* class or the *Diabetes and Hypertension*, compared to Class 1. Only the *Cardiovascular* class had an association with the locality.

Those that drank alcohol in the past 12 months were less likely to belong to the *Diabetes and Hypertension* class, compared to Class 1. Also, current smokers were more likely to belong to the *Cardiovascular* class. People with higher BMIs (overweight or obese) tended to belong to the *Cardiovascular* class or the *Diabetes and Hypertension* class, compared to Class 1.

## Discussion

We examined the profile of multimorbidity in South Africa and found that women made up 70% of the multimorbid population. Multimorbidity occurred across all age groups in our sample. This study identified four latent classes among the multimorbid in South Africa.

Nearly 40% of the multimorbid population belonged to the *HIV, Hypertension and Anemia* class. This is a significant finding, showing the large overlap of chronic infectious and NCDs in South Africa. The co-occurrence of HIV, hypertension and anemia is not well documented, although one Tanzanian study noted the high prevalence of anemia, hypertension and undernutrition among people living with HIV (PLWH). They also noted that PLWH co-morbid with anemia had higher mortality rates (27). Individually, HIV and anemia, as well as HIV and hypertension have been well studied. For example, the prevalence of anemia among PLWH is known to be high in developing countries (28). HIV infection can result in hematological complications such as anemia which ART can be beneficial for reducing, except for zidovudine-based ART regimens which worsen the condition (28). It is estimated that a quarter of PLWH also have hypertension, with the prevalence being higher in ART-experiencing patients (29). HIV and hypertension tend to be comorbid due to traditional hypertension risk factors, HIV-specific factors and the effect of ART (30). The disease cluster *HIV, Hypertension and Anemia* need further exploration to determine whether it is preventable, how ART regimens affect the development of these diseases and how it affects adherence to ART and quality of life for PLWH.

Approximately, 24% of the multimorbid population belonged to the *Anemia and Hypertension* class. This disease combination was also identified in a rural South African cohort (31). However, why these diseases co-occur is less well understood. A study investigated the relationship between hypertension, anemia and pulse pressure using the Korea National Health and Nutrition Examination Survey and found that while pulse pressure and hypertension were related; the relationship between anemia and hypertension was confounded by waist measurement and BMI (32). This is another disease cluster requiring further investigation.

The remaining two disease classes are commonly recognized as being co-morbid. The *Cardiovascular* class members had high probabilities of hypertension, with moderate probabilities of high cholesterol, heart disease and diabetes. The smallest class (17%) in the multimorbid population was *Diabetes and Hypertension*. Diabetes and hypertension share common etiologies and disease mechanisms (e.g., genetic and environmental factors, obesity, inflammation, oxidative stress, insulin resistance and physical activity) (33). Though these disease combinations are more well-known, whether people are receiving appropriate integrated care for these disease conditions remains a concern.

We also considered the influence of sociodemographic variables and lifestyle risk factors on class membership. Tobacco smokers were more likely to belong to the *Cardiovascular-related* class which makes sense given that smoking is a risk factor for many diseases in that class. Smoking may have also been a factor in the *Diabetes and Hypertension* class, but the findings were not significant, possibly due to the small sample size. The effect of alcohol consumption was less clear, but it appears alcohol consumption may have been common in the reference class (which was likely younger than the other classes due to the dominance of HIV). Being either overweight or obese increased the probability of belonging to the *Cardiovascular* and *Diabetes and Hypertension* classes. This is also realistic given that the members of the classes were dominated by HIV and anemia and may have been slimmer.

Our study is one of a few studies to use LCA to determine multimorbidity patterns in the South African general population and to our knowledge, is the only study to include chronic infectious diseases such as HIV and TB. We also included younger people in our analysis which is useful in investigating disease burden across different age groups. The data used in this analysis was limited to the available data from the original survey and the small number of included disease conditions may have affected the results. The analysis included certain self-reported disease conditions (e.g., bronchitis/COPD, heart disease, high cholesterol, stroke and TB); which may have led to under-reporting as people could be unaware that they have a disease. The SADHS 2016 did not collect data on adult mortality which is also a limitation of the analysis. If people with specific disease patterns have higher mortality rates, this could affect the prevalence estimates.

To our knowledge, two studies have used LCA locally to determine common disease clusters (14, 34). Chidumwa et al. (14) analyzed the 2014/2015 Study on global AGEing (SAGE) in people 45 years and older. The LCA identified three classes: (a) minimal multimorbidity risk (83%), (b) concordant multimorbidity (hypertension and diabetes−11%) and (c) discordant multimorbidity (angina, asthma, chronic lung disease, arthritis, and depression−6%). Their study found that female participants and older adults were more likely to belong to concordant multimorbidity groups. On the other hand, tobacco users had a high probability of belonging to the discordant multimorbidity group. Bayes-Marin et al. (34) used baseline SAGE data in people aged 50 years and older and grouped South Africa with Ghana to represent the African region. The LCA identified three classes: (a) *cardio-metabolic* class, (b) *respiratory-mental-articular* class, and (c) *healthy* class. Their study found that physical activity was protective against multimorbidity, and that smoking was a risk factor for the “respiratory-mental-articular” group of disease. Our study differed from both studies in several ways—we excluded people without multimorbidity, we included younger individuals, and we included chronic infectious disease conditions such as HIV. The sex and lifestyle risk factors associated with disease class membership is difficult to compare between these studies, due to the varying disease classes identified. However, our study was similar in finding that age, sex, and smoking were related to disease class membership.

## Conclusion

Our findings are important for service delivery in South Africa and affirm that integrated care is immediately needed. The largest multimorbid disease class identified in this study was HIV, hypertension and anemia—a mixture of chronic infectious diseases and NCDs. This overlap of chronic infectious and NCD burden highlights that HIV cannot be treated in a silo. The burden of hypertension needs to be addressed urgently. Hypertension was present in our multimorbid population, even at young ages. While South Africa has policies for the major NCD risk factors (tobacco smoking, unhealthy diets, harmful use of alcohol and physical inactivity), implementation is a problem (35). We have also shown that lifestyle risk factors are associated with disease class membership and reduction in these factors may help prevent future epidemics of multimorbidity.

We have highlighted disease clusters that are not well-researched. More studies are needed to assess whether disease associations are spurious, a result of interactions or similar biological pathways. We also need an understanding of whether these diseases are related to medication and whether alternative medication could avert the co-occurrence of disease. These disease clusters could also be used to advocate for better screening and management of patients. Analyses such as these could be important for policy formation in the country (e.g., to create policies targeting the healthcare of older adults). It can also be used to champion integrated health services, especially around the identified disease patterns.

Lastly, we have shown that HIV is an important part of multimorbidity in South Africa. Where possible, it should be included in research. Just as service delivery should not occur in a silo, research also needs to be integrated to uncover realities on the ground.

## Data availability statement

Publicly available datasets were analyzed in this study. This data can be requested from the DHS Program using the following URL: https://dhsprogram.com/methodology/survey/survey-display-390.cfm.

## Ethics statement

The studies involving human participants were reviewed and approved by participants gave their consent to take part in the survey, take measurements of heights, weights and blood pressure and collect blood specimens. This study was a secondary data analysis of an anonymised dataset which was obtained from the DHS programme. Ethics clearance to conduct this study was granted by the Biomedical Research Ethics Committee of the University of the Western Cape (BM20/5/8) as part of the author's Ph.D. project. Written informed consent to participate in this study was provided by the participants' legal guardian/next of kin.

## Author contributions

RR, BW, AC, and VP conceptualized the manuscript. Data analysis was conducted by RR and overseen by AC. RR drafted the first version of the manuscript. All authors contributed to revising the manuscript and approved the final version.
