# Supplementary material for: Multimorbidity patterns in South Africa: A latent class analysis
Source: Front Public Health. 2023 Jan 11;10:1082587. doi: 10.3389/fpubh.2022.1082587 (PMC9875075; doi:10.3389/fpubh.2022.1082587)
Supplement: Supplementary file 1 [file Table_1.DOCX]

Supplementary Material

**S1 Table. Additional data cleaning details for disease conditions, sociodemographic details and behavioural factors**

| **Variable** | | **Survey Question / Description of data collection** | **Notes on data cleaning** | **Coding** |
| --- | --- | --- | --- | --- |
| **DISEASES** | | | | |
| **Diseases (self-reported)** | **Diabetes*** | Has a doctor, nurse or health worker told you that you have or have had any of the following conditions: diabetes or blood sugar? | None. | - No=0 - Yes=1 |
|  | **Emphysema/ Bronchitis/ COPD** | Has a doctor, nurse or health worker told you that you have or have had any of the following conditions: chronic bronchitis, emphysema, or COPD? | None. | - No=0 - Yes=1 |
|  | **Heart disease** | Has a doctor, nurse or health worker told you that you have or have had any of the following conditions: Heart attack or angina/chest pains? | None. | - No=0 - Yes=1 |
|  | **High blood cholesterol** | Has a doctor, nurse or health worker told you that you have or have had any of the following conditions: high blood cholesterol or fats in the blood? | None. | - No=0 - Yes=1 |
|  | **Stroke** | Has a doctor, nurse or health worker told you that you have or have had any of the following conditions: stroke? | None. | - No=0 - Yes=1 |
|  | **TB in the last 12 months** | Has a doctor, nurse or health worker ever told you that you have TB? | Two questions were combined. ‘No’ refers to people who have not had TB or had TB more than 12 months ago. ‘Yes’ refers to people who had TB in the last 12 months. | - No=0 - Yes=1 (TB in the last 12 months) |
|  |  | When was the last time you had TB? |  |  |
| **Diseases (biomarkers)** | **Diabetes (HbA1c)*** | Nurses collected blood specimens from finger pricks. If the participant consented to both HbA1c and HIV testing, five blood spots were collected on filter paper card and a unique barcode was affixed to the card. The dry blood specimens (DBS) were analysed with a blood chemistry analyser which measured total haemoglobin concentration by a colorimetric method. A more detailed description can be found in the SADHS 2016 Report (p.5), including the adjustment of results (p.271) ([1](#_ENREF_1)). | Data cleaning followed the procedure used for the second South African Comparative Risk Assessment Study.([2](#_ENREF_2)) Inconclusive HbA1c values were eliminated.  Diabetic status was assigned if HbA1c >=6.5 mmol. Those on medication to control diabetes were also assigned diabetic status. | - No= 0 - Yes=1 (has diabetes) |
|  | **HIV** | If the participant consented to both HbA1c and HIV testing, five blood spots were collected on filter paper card and a unique barcode was affixed to the card. All samples were tested with an enzyme-linked immunosorbent assay (ELISA), the Genscreen HIV 1/2 Combi Assay (Bio-Rad). If the first ELISA was positive, a second ELISA was done (ELISA 2), the E411 Cobas HIV 1/2 Combi Assay (Roche). A more detailed description can be found in the SADHS 2016 Report, p.5 ([1](#_ENREF_1)) | The results of the first HIV assay was taken as confirmation of HIV. | - No=0 - Yes=1 (has HIV) |
| **Diseases (biomarkers cont.)** | **Anaemia** | Nurses collected blood samples were drawn from a finger prick and collected in a microcuvette. The analysis of haemoglobin was conducted on site using a battery-operated portable HemoCue 201+ analyser. The results were adjusted for smoking status and altitude. (SADHS 2016 Report, p. 5). | Anaemia was coded in the DHS 2016 as: None, mild, moderate and severe. According to the DHS-7 Standard Recode Manual ([3](#_ENREF_3)), anaemia levels below 7.0 g/dl are considered as severe anaemia. Moderate anaemia is considered levels between 7.0g/dl and 9.9g/dl. For pregnant women, mild anaemia are levels between 10.0 g/dl and 10.9 g/dl and between 10.0 g/dl and 11.9 g/dl for all other adult women. These categories were recoded to anaemia present (mild, moderate, severe) or absent. | - No=0 - Yes=1 (has mild, moderate or severe anaemia) |
|  | **Hypertension** | Three readings were taken using Omron 1300 digital blood pressure monitors. Measurements were taken at three-minute intervals. The 2016 SADHS took the third measurement to classify the participant with hypertension according to the WHO 1999 categories. A more detailed description can be found in the SADHS 2016 Report, p.5 [38] | The data cleaning followed the procedure used in the second South African Comparative Risk Assessment Study([4](#_ENREF_4)) and was in agreement with the procedure applied by [Zhou, Bentham (5)](#_ENREF_5). The first measurement was excluded and the average of the remaining replicated readings was considered as the subject’s blood pressure. If one value was missing of the two readings, the observation was excluded.  Replicated measurements of systolic and diastolic blood pressure were assessed for the presence of implausible values (systolic BP < 70 mm Hg or > 270 mm Hg, diastolic BP < 30 mm Hg or > 150 mm Hg), which were set to missing. People on medication were included in those that had hypertension.  Categories[[19](#_ENREF_19)] were defined as follows:   - Hypertension absent: Systolic < 120 *mmHg* & diastolic < 80 *mmHg* - Pre-hypertension: Systolic: 120–139 *mmHg* or diastolic: 80-89 *mmHg* - Stage 1 Hypertension: Systolic: 140–159 *mmHg* or diastolic: 90–99 *mmHg* - Stage 2 hypertension: Systolic ≥160 *mmHg* or diastolic ≥100 *mmHg*   Hypertension was coded as absent or present (stage 1 or stage 2 hypertension). | Hypertension:   - No=0 - Yes=1 (has hypertension stage 1 or 2)   Hypertension categories:   - Normal=0 - Pre-hypertension=1 - Stage 1 hypertension =2 - Stage 2 hypertension=3 |
| **Disease (combination)** | **Self-reported diabetes & biomarker diabetes** |  | The results of the self-reported diabetes and HbA1c diabetes were combined. | Self-reported diabetes / biomarker diabetes:   - No= 0 - Yes=1 (has diabetes) |
| **SOCIODEMOGRAPHIC AND LIFESTYLE FACTORS** | | | | |
| **Self-report** | **Educational attainment** | What is the highest grade or form you completed at that level? | Responses were divided into three categories: Primary or less, Secondary and Tertiary. | - Primary or less=0 - Secondary complete=1 - Tertiary=2 |
|  | **Wealth index** | *Various questions.* | The 2016 SADHS wealth index scores households according to the types of goods that are owned and other characteristics. | - Quintile 1 (Poorest)=0 - Quintile 2 (Poorer)=1 - Quintile 3 (Middle)=3 - Quintile 4 (Richer)=4 - Quintile 5 (Richest)=5 |
|  | **Current alcohol use** | Have you ever consumed a drink that contains alcohol such as beer, wine, ciders, spirits, or sorghum beer? | Responses to the two questions were combined. “Yes” refers to participants who drank alcohol in the past 12 months and “No” refers to participants who have not drank alcohol or drank more than 12 months ago. | - No=0 - Yes=1 |
|  |  | Was this within the last 12 months? |  |  |
|  | **Current tobacco smoker** | Do you currently smoke tobacco every day, some days, or not at all? | “Yes” represents participants who smoke daily or occasionally. “No” represents participants who do not smoke. | - No=0 - Yes=1 |
| **Measured** | **Body Mass Index (BMI)** | Height was measured with a Seca 213 portable stadiometer, and weight was measured with a Seca 878 digital scale. A more detailed description can be found in the SADHS 2016 Report [38] | The data cleaning followed the procedure used in the second South African Comparative Risk Assessment Study ([6](#_ENREF_6)). Implausible values were converted to missing values. Height in centimeters was calculated. The BMI was calculated using the *BMI* STATA package.  **BMI category (BMI (kg/m^2^) range:**   - Underweight (15 .0 - <18.5) - Normal weight (18.5 - <25.0) - Overweight (25.0 - <30.0) - Obesity grade 1 (30.0 - <35.0) - Obesity grade 2 (35.0 - <40.0) - Obesity grade 3 (40.0 - <60.0) | BMI categories:   - Underweight=0 - Normal weight=1 - Overweight=2 - Obesity grade 1=3 - Obesity grade 2=4 - Obesity grade 3=4 |

**Fig S1. Estimated disease prevalence among multimorbid people, by disease condition and age group (weighted data)**

**Table S2. Estimated disease prevalence among multimorbid people, by disease condition and sex (weighted)**

| **Disease condition** | **Prevalence**  **% (95% CI)** | |
| --- | --- | --- |
|  | **Male** | **Female** |
| Hypertension | 83.8 (79.5 - 87.4) | 77.8 (74.4 - 80.8) |
| Anaemia | 48.5 (43.5 - 53.5) | 53.9 (50.5 - 57.4) |
| HIV | 39.9 (34.8 - 45.3) | 47.8 (44.4 - 51.3) |
| Diabetes | 35.7 (30.9 - 40.7) | 35.1 (32.0 - 38.4) |
| High cholesterol | 16.1 (12.5 - 20.6) | 12.6 (10.3 - 15.4) |
| Heart disease | 12.1 (9.2 - 15.6) | 12.5 (10.6 - 14.6) |
| Bronchitis/COPD | 5.6 (3.8 - 8.2) | 5.6 (4.1 - 7.4) |
| Stroke | 5.0 (3.1 - 7.8) | 5.6 (4.4 - 7.1) |
| TB in past 12 months | 4.1 (2.7 - 6.3) | 4.5 (3.2 - 6.4) |

**Table S3. Membership probabilities and standard errors for each latent class.**

| **Class name** | | **Membership probability** | **Standard errors** |
| --- | --- | --- | --- |
| **1** | **HIV, Hypertension & Anaemia** | 0.394 | 0.02 |
| **2** | **Anaemia and Hypertension** | 0.237 | 0.02 |
| **3** | **Cardiovascular** | 0.199 | 0.02 |
| **4** | **Diabetes and Hypertension** | 0.170 | 0.01 |

**Table S4. Item response probabilities and standard errors by disease condition for each class.**

| **Class name** | | **Disease condition item response probabilities**  **(standard errors)** | | | | | | | | | |
| --- | --- | --- | --- | --- | --- | --- | --- | --- | --- | --- | --- |
|  |  | **Anaemia** | **Bronchitis/**  **COPD** | **Diabetes** | **Heart disease** | **High cholesterol** | **HIV** | **Hypertension** | **Stroke** | **TB** |  |
| **1** | **HIV, Hypertension and Anaemia** | 0.594 (0.025) | 0.012 (0.006) | 0.103 (0.017) | 0.051 (0.011) | 0.009 (0.007) | 1.000 (0.000) | 0.605 (0.027) | 0.018 (0.005) | 0.075 (0.013) |  |
| **2** | **Anaemia and Hypertension** | 1.000 (0.000) | 0.025 (0.010) | 0.309 (0.027) | 0.079 (0.019) | 0.044 (0.021) | 0.001 (0.000) | 0.870 (0.021) | 0.032 (0.011) | 0.018 (0.012) |  |
| **3** | **Cardiovascular** | 0.167 (0.056) | 0.224 (0.034) | 0.346 (0.046) | 0.375 (0.038) | 0.502 (0.046) | 0.084 (0.037) | 0.938 (0.017) | 0.175 (0.026) | 0.047 (0.014) |  |
| **4** | **Diabetes and Hypertension** | 0.002 (0.000) | 0.001 (0.006) | 0.999 (0.000) | 0.059 (0.021) | 0.144 (0.034) | 0.088 (0.029) | 0.999 (0.001) | 0.028 (0.018) | 0.006 (0.006) |  |

**Table S5. Latent class model with covariates: membership probabilities** **and standard errors.**

| **Class name** | | **Membership probability** | **Standard errors** |
| --- | --- | --- | --- |
| **1** | **HIV, Hypertension & Anaemia** | 0.390 | 0.013 |
| **2** | **Anaemia and Hypertension** | 0.167 | 0.010 |
| **3** | **Cardiovascular** | 0.183 | 0.019 |
| **4** | **Diabetes and Hypertension** | 0.259 | 0.019 |

**Table S6. Latent class model with covariates: Item response probabilities and standard errors.**

| **Class name** | | **Disease condition item response probabilities**  **(standard errors)** | | | | | | | | | |
| --- | --- | --- | --- | --- | --- | --- | --- | --- | --- | --- | --- |
|  |  | **Anaemia** | **Bronchitis/**  **COPD** | **Diabetes** | **Heart disease** | **High cholesterol** | **HIV** | **Hypertension** | **Stroke** | **TB** |  |
| **1** | **HIV, Hypertension and Anaemia** | 0.604 (0.024) | 0.010 (0.004) | 0.057 (0.015) | 0.051 (0.011) | 0.005 (0.004) | 0.990 (0.007) | 0.597 (0.028) | 0.016 (0.005) | 0.082 (0.014) |  |
| **2** | **Anaemia and Hypertension** | 0.999 (0.000) | 0.031 (0.011) | 0.001 (0.000) | 0.090 (0.025) | 0.054 (0.023) | 0.003 (0.002) | 0.956 (0.018) | 0.025 (0.010) | 0.001 (0.002) |  |
| **3** | **Cardiovascular** | 0.146 (0.042) | 0.188 (0.037) | 0.351 (0.086) | 0.373 (0.050) | 0.490 (0.056) | 0.099 (0.036) | 0.956 (0.016) | 0.163 (0.029) | 0.038 (0.016) |  |
| **4** | **Diabetes and Hypertension** | 0.333 (0.040) | 0.019 (0.009) | 0.999 (0.000) | 0.051 (0.018) | 0.066 (0.047) | 0.133 (0.029) | 0.871 (0.025) | 0.024 (0.010) | 0.007 (0.005) |  |

# References

1. National Department of Health, Statistics South Africa, South African Medical Research Council, and ICF. South Africa Demographic and Health Survey 2016 Pretoria, South Africa, and Rockville, Maryland, USA: NDoH, Stats SA, SAMRC, and ICF (2019). Available from: <https://dhsprogram.com/pubs/pdf/FR337/FR337.pdf>.

2. Pillay van Wyk V, Cois A, Kegne A, Roomaney R, Levitt N, Turuwa E, et al. Estimating the Changing Disease Burden Attributable to High Fasting Plasma Glucose in South Africa for 2000, 2006 and 2012 *S Afr Med J* (2022) In Press. doi: NA.

3. ICF. Demographic and Health Surveys Standard Recode Manual for Dhs7 Rockville, Maryland, U.S.A.: ICF: The Demographic and Health Surveys Program. (2018). Available from: <https://dhsprogram.com/pubs/pdf/DHSG4/Recode7_DHS_10Sep2018_DHSG4.pdf>.

4. Nojilana B, Peer N, Abdelatif N, Cois A, Schutte AE, Labadarios D, et al. Estimating the Changing Burden of Disease Attributable to High Blood Pressure in South Africa for 2000, 2006 and 2012. *S Afr Med J* (2022) In Press. doi: NA.

5. Zhou B, Bentham J, Di Cesare M, Bixby H, Danaei G, Cowan MJ, et al. Worldwide Trends in Blood Pressure from 1975 to 2015: A Pooled Analysis of 1479 Population-Based Measurement Studies with 19.1 Million Participants. *Lancet* (2017) 389(10064):37-55. doi: 10.1016/S0140-6736(16)31919-5.

6. Bradshaw D, Joubert JD, Abdelatief N, Cois A, Turawa EB, Awotiwon OF, et al. Estimating the Changing Burden of Disease Attributable to High Body Mass Index in South Africa for 2000, 2006 and 2012. *S Afr Med J* (2022):583-93. doi: 10.7196/SAMJ.2022.v112i8b.16488.
